# Faculty of Comparative Medicine and Veterinary Science, McGill University, Montreal

**Published:** 1890-05

**Authors:** 


*Faculty of Comparative Medicine and Veterinary Science, McGill University, Montreal.*—The annual public meeting of Convocation for the conferring of degrees in Comparative Medicine and Veterinary Science was held in the William Molson Hall on Tuesday, April 1st, at 3 P.M.

The members of Convocation met in the library at half-past two. At 3 o'clock precisely the procession was formed and made its way up to the platform from the library entrance. The following was the order observed: The visitors; the president and chancellor; the remaining governors, in order of appointment; the principal and vice-chancellor; the vice-principal; the remaining fellows, in order of first appointment; the professors of the college and university, in order of appointment; those of affiliated colleges, members of convocation, in like order; the secretary, registrar and bursar; the lecturers, tutors and other instructing officers of the college and university; members of convocation, in like order; those of affiliated colleges, members of convocation, in like order; doctors of divinity; doctors of laws or of civil law; doctors of medicine; bachelors of divinity; masters of arts; masters of engineering and applied science; bachelors of civil law; bachelors of arts; bachelors of applied science, and graduates in civil engineering.

The robed procession streamed over the platform and occupied seats in the appointed places. Sir Donald A. Smith, the chancellor, presided, and at his left was the principal, Sir William Dawson. Around him were the governors, Mr. John H. R. Molson, Mr. W. C. MacDonald, Mr. Samuel Finley, Alexander Johnson, LL.D., dean of the faculty of arts; Dr. Craik, dean of the medical faculty, and Dr. Ross, the vice-dean; Prof. Bovey, dean of the faculty of applied science; N. W. Trenholme, Q.C., dean of the faculty of law; Dr. McEachran, dean of the faculty of comparative medicine; Dr. Stewart, Dr. Shepherd, Dr. Wilkins, Dr. Cameron, Dr. Mills, Dr. Girdwood, Dr. Gardner, Dr. Rodger, Dr. Grant, Dr. Birkett, Dr. Johnston, Dr. Finley, Dr. Ruttan, Dr. Baker, Dr. McEachran, Rev. Principals MacVicar, Shaw and Barbour, Prof. Penhallow, F. W. Kelley, Ph.D., Prof. J. Clarke Murray, John Dougall, M.A., Prof. C. E. Moyse, Rev. Dr. Cornish, Rev. Prof. Scrimgour, Mr. Justice Cross, Rev. Prof. Coussirat, Dr. J. W. Gadsden, Philadelphia; J. W. Brackenridge, B.C.L.; W. Skaife, B A. Sc., P. Toewes, M.A.; E H. Hamilton, B.A. Sc.; J. A. MacPhail, B.A.; M. W. Hopkins, B. A. Sc.

Rev. Dr. Cornish opened the proceedings with the usual form of prayer.

As a preliminary to the exercises of the faculty of comparative medicine, the degree of D.V.S. was conferred upon Dr. McEachran, the dean. He then submitted his report, as follows, giving the prize honors, and pass list:—

First prize and medal, the gift of the Council of Agriculture, awarded to R. N. Walsh, Huntingdon, Quebec, for best examinations in all branches during three years.

*Practice of Medicine and Surgery.*—1st, L. E. Willyoung; 2d, J. F. Scott.

*Veterinary Obstetrics.*—1st, R. N. Walsh; 2d, L. E. Willyoung.

*Anatomy.*—1st, J. F. Scott; 2d, L. E. Willyoung.

*Chemistry.*—1st, Twombley.

*Physiology.*—1st, G. Macauley.

*Materia Medica.*—1st, Twombley; 2d, Macauley.

*Botany.*—1st, J. W. Moffat.

*Botany.*—First Year.—Moffatt, (J. W.), Plaskett, Dyer, Wells, Perly, Moffatt (S. J.), Seale, Lofgren, Lee, Gangloff, Barton, Dunton, MacDougall, Robertson.

*Histology.*—First Year.—First-class Honors.—Twombley, J. W. Moffatt, Plaskett.

Second-class.—Robertson and Dyer.

Third-class.—Bolger, Gangloff, MacDougall, McDonald, Rathbone, Comstock, Lee, S. J. Moffatt, McNaughton, Robb, Pote, Seale, and Wells.

*Physiology.*—Second Year.—First-class, over 75 per cent—Macauley and Twombley.

Second-class, over 66 per cent—Hayman.

Third-class.—McCrank, McDonald, Sturrock, Comstock, St. Louis, Cannon, Gorham, Ramsay, Townsend, Watson, D. M. McDonald, Higginson, Miller, and Simpson.

*Chemistry.*—Second Year.—Twombley, McCrank, Comstock, Sturrock,

St. Louis, Crossman, Simpson, Watson, Cannon, T. B. McDonald, Townsend, and Macauley.

*Materia Medica.*—Second Year.—Honors.—Twombley, Macauley, Sturrock, Higginson, D. McDonald, R. A. Ramsay, Simpson, J. B. McDonald, Watson, McCrank, Comstock.

*Pass.*—Townsend, McNaughton, Cannon, Dimton, Henderson, Gorham, Miller, St. Louis.

*Practice of Medicine and Surgery.*—Final Year.—Willyoung, Scott, Scanlan, McGlue, Walsh, Hayman, Darling, Crossman.

*Anatomy.*—Final Year.—Pass List.—Scott, Willyoung, Hayman, Scanlan, Walsh, Darling, McGlue, and Crossman.

*Pathology.*—Final Year.—Willyoung, Crossman, Walsh, McGlue, Scanlan, Scott, Darling, Hayman, and Barber.

*Veterinary Obstetrics and Diseases of Cattle.*—Walsh, Willyoung, Scott, Darling, Scanlan, Hayman, Crossman, and McGlue.

The Degree of Doctor of Veterinary Science was then conferred, first, upon T. Wesley Mills, M.A., M.D., and upon Dr. C. E. McEachran and Dr. C. M. Baker. Following them came the other graduates.

Mr. Scanlan then read the valedictory for the graduates in comparative medicine as follows:

Mr. Chancellor, etc.:—My first words to-day shall be those of joy and gladness. In the name of the Faculty of Comparative Medicine, I invite you all, most cordially, to join in celebrating its first birthday, and in offering to Dear Old Mother McGill our heartiest congratulations.

This day upon which *we*, as students of comparative medicine, appear here for the first time—this day, I say, marks the dawn of a new era for our institution.

The heretofore Montreal Veterinary College, after incessant hard work and conscientious labor, has succeeded in rendering its merits so conspicuous that it has drawn to itself the attention of the professors of McGill University—has earned their approbation and interested them in its career. It is to these gentlemen that our faculty now owes its present enviable position; and were any pleading necessary to establish its merit and its worth, we would only say that men of their knowledge paid it, perhaps, the greatest compliment within their reach by placing it among the faculties of this great university.

When we entered our college, three years ago, we scarcely hoped that we should be the first to be honored with a university degree. Time will, we think, never efface the pleasant recollections of this day. When this college was founded, some twenty-four years ago, it had as its sole patron Dr. McEachran, to our minds the most earnest and zealous member of the profession. At the beginning our number was small, and to-day such is still the case.

Let me say this redounds all the more to the credit of our institution, which has ever remained faithful to the good principles she upheld at her birth, and that if, in point of numbers, her class was and is still small, yet she can replace quantity by quality, and point with pride to the fact that her graduates now occupy the most important positions in whatever country they may be, and reflect credit on the house that sent them forth. If one were to look for a cause productive of the good effect of which I have just spoken, one might point out the comparatively severe, but absolutely wise, system of education which has always been in vogue in our institution.

Two years are elsewhere considered sufficient to prepare a man for the exercise of his profession; with us three are thought and are known to be not too many. These two doctrines are still in conflict upon our continent, but the past justifies the present, and gives us reason to hope that some future day the serious system of education adopted at Montreal may be everywhere accepted as the only true and rightful one.

As a claim to the attention as well as to the gratitude of the public, we might say that it is to our profession that the people of Canada and the United States are indebted for the non-prevalence of those scourges which to-day ravage Europe, causing losses oftentimes enormous.

With a view to discuss those diseases, to cause them to be more easily vanquished and better understood, veterinary medical associations have been formed; one of the first founded in this country was established in connection with our college.

In the last few years a psychological society was founded by Dr. Mills, and it has gone far in pleading the cause of the dumb brute; the future, we trust, will show the good it has done.

Now, you will pardon us if we say a few words in our own behalf. There was a time, not long ago, when a veterinary surgeon had the unenviable reputation of being an ignorant man; when he was placed, even by enlightened people, far below themselves on the ladder of social and intellectual life.

That time, we believe, has passed away, and if some remnant of that ignorance still exists, we feel that it will soon disappear before the criticism and condemnation of an intelligent public.

Our graduates of former years had great responsibilities resting on their shoulders. Upon them devolved the task of upholding through life their own personal honor and that of the faculty to which they belonged. Upon us weighs a greater charge, or rather the greater privilege of defending the rights of the University, of ever working with this end in view to honor her for the inestimable favor she has conferred upon us this afternoon.

The immense benefit we will derive from the promotion of our college will, to a great extent, depend upon our future conduct; our aspirations must ever be onward, and we should keep in view the fact that all we do to advance our profession will shed its lustre on old McGill.

And now one word more and I have done.

It has been truly said that life is made up of meetings and partings. The former are oftentimes joyous, the latter are nearly always sad. However, at this final moment, when we are about to leave the professors whose devotion and self-sacrifice have always—have ever been ours, and the students whose friendship has caused us many joyous hours—at this moment, I say, we draw consolation and strength from the knowledge that one and all, you will tender us your best wishes, and bid us success and happiness in our journey through life.

THE REPLY TO THE GRADUATES.

Dr. McEachran, Dean of the Faculty, gave the address to the graduates in comparative medicine. Before addressing himself specially to the graduates, he referred to the branches of scientific study to which his faculty was devoted, and traced the rise of veterinary science from the earliest time, until now it is competent to rank side by side with human medicine. It embraced the study and comparison of the anatomy, physiology and diseases of animals with those of the highest type of animal—man. Of necessity the study was confined more particularly to domestic animals, in which field of study they had a most comprehensive one, when it was considered that medical science, in all its collateral branches and subdivisions of study, had to be considered in relation to the different classes of domestic animals. Looked at in a still broader light, when we consider that in Canada alone there are about 1,165,288 horses and 3.866,479 cattle and other horned animals, worth in the aggregate, say $200,000,000, and representing no small proportion of the country's wealth, and knowing, as we do, that every one of these animals is, like ourselves, liable to accidents and diseases, many of which are preventable, many curable, surely the ministers to these animals in sickness, from a pecuniary point alone, ought to be men well grounded in medical science. Day by day the sciences of human and comparative medicine were becoming more closely united and more and more dependent upon one another. The day was not far distant when a course of comparative medicine would be found a part of the curriculum of every medical school. He next paid a glowing eulogy to McGill College, which had done so much for learning throughout the country.

Speaking to the graduates, he said: Gentlemen—You have now completed the curriculum prescribed for you, and have been admitted to the degree of doctor of veterinary science. This you have gained after a hard course of study, and having passed most searching written and oral tests, by independent examiners appointed by the government, as well as the examinations prescribed by the university.

Presumably, therefore, you are qualified to practice; I believe you to be so. Your examiners declare you so, this great university announces you so. Now, gentlemen, do not imagine this memorable day in your lives is to mark the end of your studies; by no means.

During your pupilage you have had but little time to familiarize yourselves with the literature of your profession other than your text-books. It will now be your duty to do so. To keep pace with the rapidly-evolving science of medicine you must be constant readers of scientific journals and new publications, not alone those directly bearing on your own particular profession or science, but on all collateral subjects. There is no profession which requires in its members so varied a store of general knowledge as does yours.

In the daily walks of life you will come in contact with all classes of society, and all degrees of rank and station. You will be expected to have a general knowledge more or less extensive, and be expected to engage in intelligent conversation on almost every conceivable subject which happens to be of particular interest to each special client. Thus, the advanced farmer leads you into discussions on "silos," on feeding standards, the relative merits of this or that breed of cattle, horses, sheep, or swine.

The sporting man imagines that you should know all winners and pedigrees of noted horses. The politician expects you to be thoroughly conversant with the bills before the house, and will be surprised if you admit that you do not wade through Hansard regularly.

It is your duty, therefore, to be a student of everything around you; be observant, and gather information from every source possible. Make it a habit, on every occasion when you are asked for information which you cannot impart from want of knowledge, to note it down, and go to your library and inform yourselves of it for future use. So in your practice acquire a habit of noting cases, record every case of more than passing interest, and study the subject carefully, read every available standard author on it, and in the light of knowledge so obtained, applied to the case under observation, you will soon become masters of your profession.

Never miss an opportunity of making a post-mortem examination; nothing aids a man so much in making a correct diagnosis as the repeated corrections and errors disclosed by a post-mortem examination. Never waste a pathological specimen; think how much good others may gain who succeed you as students of comparative medicine, from even one specimen, accompanied by a carefully recorded history. Museum specimens, accompanied by histories, are of great service in illustrating didactic lectures.

In your practice acquire the habit of careful clinical inspection, and ever remember that your patients, though dumb, are in all things like as we are—they hear, see, feel, smell, taste, suffer pain, and enjoy pleasureable emotions just as we do. Deal with them in the full consciousness of these facts. Do not frighten them either by voice or look, never cause even the slightest pain that you can prevent, and never nauseate them by nauseous medical compounds such as you would consider barbarous in a doctor to prescribe for yourself. In surgical operations, don't forget the sentient nerves which ramify every part of the body, employ every means in your power to lessen the suffering in necessary operations; too little use is made of the valuable discoveries applied to lessen human suffering; local and general anæsthetics. In your fees be moderate—by no means undervalue your professional services—but be satisfied with fair, moderate charges. Acquire prompt business habits, keep engagements punctually; nothing drives friends and clients away as quickly as inattention to engagements. Collect your accounts regularly, pay your own debts promptly, and avoid debt as you would a quicksand. Gentlemen, in going out into the great world, do not suppose that you will not have to meet with opposition and discouragements—for you shall; but meet them manfully; and let me assure you, that with your scientific attainments, and by unimpeachable conduct, by industry, sobriety, and fair dealing with all men, you need have no fear for the future. The importance of your profession is daily becoming more and more understood; if you fail, blame not your profession, but blame yourselves, and never forget that, under no circumstances, can your profession disgrace you—but you may disgrace your profession. Choose for your companions those only who are enlightened and refined; let your reading and your conversation always be elevating in character. In all things be gentlemen; live as gentlemen, talk as gentlemen, and dress like gentlemen. Much more might profitably be said on your duty to yourselves, to your clients, to your profession, and to your Alma Mater, but time forbids.

In conclusion, therefore, gentlemen, on behalf of your teachers who, we trust, you will consider your lifelong friends, I say you "God-speed;" we send you forth into a wide field of scientific usefulness, in which we trust some of you, at least, will become eminent and successful men, honored and respected by your fellows and confreres. We will watch your progress as fathers do their children, and never forget that we look to you, who are the first University graduates of this faculty, to uphold the reputation of your faculty, and this great University, of which it forms a minor part.

In the name of the faculty, I beg to tender our thanks to the Provincial Government for their liberality in continuing to give us the annual grant, and to the Hon. Acting Commissioner of Agriculture, for taking the trouble to honor us with his presence to-day; to those gentlemen who constitute the board of examiners, who have travelled long distances in order to assist us, and to this great assembly, for your patience in listening to these remarks.

The Principal expressed regret that Hon. D. A. Ross, who it was expected would be present, was unable to attend. Rev. Principal MacVicar pronounced the benediction, and the convocation for 1890 was at an end.

